# Supplementary material for: Identification of pharmacological inducers of a reversible hypometabolic state for whole organ preservation
Source: eLife. 2024 Sep 24;13:RP93796. doi: 10.7554/eLife.93796 (PMC11421850; doi:10.7554/eLife.93796)
Supplement: Supplementary file 2. [file elife-93796-supp2.docx]

**Supplemental Synthesis Description**

**Step 1: Synthesis of intermediate *N,N*-diethyl-4-formylbenzamide**

4-formylbenzoic acid (10 g, 66.61 mmol) and HATU (30.39 g, 79.93 mmol) were dissolved in DMF (500 mL) at 15 °C under N_2_. The mixture was then warmed to 30 °C and stirred for 1 h. At this juncture, the mixture was cooled to 15 °C and DIEA (34.43 g, 266.43 mmol, 46.41 mL) and diethylamine (7.31 g, 99.91 mmol, 10.29 mL) were added dropwise together. The reaction was allowed to warm to rt and stirred for 12 h. The reaction mixture was diluted with H_2_O (2000 mL), and then extracted with ethyl acetate (5000 mL x 3). The combined organic layers were washed with brine (1000 mL), dried over Na_2_SO_4_, filtered, and concentrated under reduced pressure to give a residue. The residue was purified by column chromatography (SiO_2_, Petroleum ether/Ethyl acetate = 3/1 to 1/1) to give *N,N*-diethyl-4-formylbenzamide (57 g, 277.71 mmol, 83.38% yield) as a yellow solid. LC-MS m/z = 206.1 [M+H]^+^.

**Step 2: Synthesis of intermediate *N,N-*diethyl-4-[hydroxy(3-methoxyphenyl)methyl]benzamide**

To a solution of *N,N*-diethyl-4-formylbenzamide (8 g, 38.98 mmol) in THF (320 mL) was dropwise added bromo-(3-methoxyphenyl)magnesium (1 M, 46.77 mL) at 0 °C under N_2_. The reaction was stirred at 20 °C for 2 h, at which time it was diluted with H_2_O (1000 mL), and then extracted with dichloromethane (500 mL x 3). The combined organic layers were washed with brine (300 mL), dried over Na_2_SO_4_, filtered, and concentrated under reduced pressure to give a residue. The residue was purified by column chromatography (SiO_2_, Petroleum ether/Ethyl acetate = 3/1 to 1/1) to give *N,N*-diethyl-4-[hydroxy-(3-methoxyphenyl)methyl]benzamide (6 g, 19.15 mmol, 6.14% yield) as a white solid. LC-MS m/z = 314.1 [M+H]^+^.

**Step 3: Synthesis of intermediate** **4-(chloro(3-methoxyphenyl)methyl)-*N,N*-diethylbenzamide**

To a solution of *N,N*-diethyl-4-[hydroxy-(3-methoxyphenyl)methyl]benzamide (11.75 g, 37.49 mmol) in CHCl_3_ (110 mL) was added dropwise HCl (12 M, 421.80 mL). The resulting mixture was stirred at 20 °C for 12 h, at which time it was diluted with H_2_O (50 mL), and then extracted with dichloromethane (30 mL x 3). The combined organic layers were washed with brine (30 mL), dried over Na_2_SO_4_, filtered, and concentrated under reduced pressure to give 4-[chloro-(3-methoxyphenyl)methyl]-*N,N*-diethyl-benzamide (23 g, 69.31 mmol, 92.43% yield) as a yellow oil.

**Step 4: Synthesis of WB3**

To a mixture of 4-[chloro-(3-methoxyphenyl)methyl]-*N,N*-diethyl-benzamide (23 g, 69.31 mmol) and (*2R,5S*)-2,5-dimethylmorpholine (15.97 g, 138.62 mmol) in ACN (230 mL) was added K_2_CO_3_ (19.16 g, 138.62 mmol). The resulting mixture was stirred at 80 °C for 4 days. The reaction mixture was filtered, and the mother liquor was directly concentrated. The residue was purified by column chromatography (SiO_2_, Petroleum ether/Ethyl acetate = 5/1 to 2/1). The residue was further purified by preparative-HPLC (column: UniSil 10-120 C18 70x250mm; mobile phase: [water(FA)-ACN]; gradient:20%-50% B over 20 min ) and the appropriate fractions were lyophilized. The residue was again further purified by preparative-HPLC (column: Phenomenex luna C18 (250*70mm,10 um); mobile phase: [water (FA)-ACN]; gradient:10%-50% B over 35 min) and the appropriate fractions were lyophilized to give WB3 (3.3 g, 8.03 mmol, 11.59% yield) as a white solid. LC-MS m/z = 296.1 [M+H]^+^.

^1^H NMR (400 MHz, CDCl_3_) δ = 7.47 (d, J = 7.8 Hz, 2H), 7.34 - 7.27 (m, 3H), 6.88 (dd, *J* = 2.0, 8.3 Hz, 1H), 6.75 (d, *J* = 7.6 Hz, 1H), 6.72 - 6.67 (m, 1H), 5.32 (s, 1H), 3.81 (s, 3H), 3.76 - 3.66 (m, 2H), 3.64 - 3.45 (m, 2H), 3.44 - 3.24 (m, 3H), 2.61 (dd, *J* = 2.0, 11.6 Hz, 1H), 2.57 - 2.48 (m, 1H), 1.82 (dd, *J* = 9.5, 11.6 Hz, 1H), 1.24 (br d, *J* = 0.8 Hz, 3H), 1.12 (d, *J* = 6.1 Hz, 6H), 1.06 (d, *J* = 6.3 Hz, 3H).
